# Fiber inclusion strategies in broiler diets: effects on performance, modulation of gut microbiota and sensory properties of meat

**DOI:** 10.1007/s11250-026-05022-9

**Published:** 2026-04-03

**Authors:** Margarida Jorge Farias, Cláudia Goulart de Abreu, Silvana Cavalcante Bastos Leite, Ana Sancha Malveira Batista, Daiane Félix Santiago Mesquita, Janete Gouveia de Souza, Raquel Oliveira dos Santos Fontenelle, Felipe Dilelis, Robson Mateus Freitas Silveira

**Affiliations:** 1 Center for Agricultural and Biological Sciences, Universidade Estadual Vale do Acaraú (UVA), Sobral, CE 62040-370 Brazil; 2 Universidade Federal do Rio Grande do Norte (UFRN), RN-160, Km 03, Distrito de Jundiaí, Macaíba, RN 59280-000 Brazil; 3 Department of Animal Science, “Luiz de Queiroz” College of Agriculture (ESALQ), University of São Paulo (USP), Piracicaba, SP 13418-900 Brazil

**Keywords:** Agro-industrial by-products, Cecal microbiota, Chicken meat quality, Functional ingredient

## Abstract

This study evaluated the effects of neutral detergent fiber (NDF) source and inclusion level on the performance, gut microbiota, and meat quality of slow-growing broilers. A total of 400 Naked Neck chicks were distributed across two experiments: initial (1–28 d) and growth/finishing (29–70 d). Birds were fed a control diet (CD) or diets containing an additional 3% or 6% NDF from wheat bran (WB) or guava residue (GR), resulting in the following treatments: CD, WB3%, WB6%, GR3%, and GR6%. In Experiment 1, birds fed WB3% and GR6% diets showed significantly lower body weight (BW) and weight gain (WG) compared to those fed CD (*P* < 0.05). Feed conversion ratio (FCR) was significantly higher (worse) in birds fed WB3%, WB6%, and GR6% diets relative to CD (*P* < 0.05), while no significant difference was observed between GR3% and CD (*P* > 0.05). Feed intake did not differ among treatments (*P* > 0.05). A significant interaction between NDF source and inclusion level was detected for BW, WG, and FCR (*P* < 0.05). In Experiment 2, no significant differences in performance traits were observed between CD and NDF-supplemented diets (*P* > 0.05). Carcass and meat cut yields were not affected by dietary treatments (*P* > 0.05). However, relative liver weight was significantly higher in birds fed guava residue-based diets (*P* < 0.05). Regarding sensory attributes, the GR6% diet promoted significantly higher scores for appearance and color (*P* < 0.05) and received the highest consumer preference. Microbiological analyses, although based on pooled samples, indicated a numerical trend toward lower thermotolerant coliform counts in NDF-supplemented groups. In conclusion, although NDF supplementation did not improve growth performance, the inclusion of 6% NDF from guava residue enhanced meat color and consumer preference, highlighting its potential as a functional ingredient for slow-growing broilers.

## Graphical Abstract

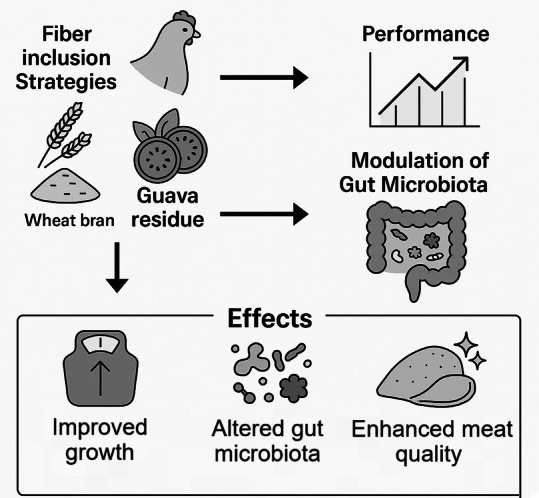

## Introduction

Alternative fiber-rich feeds are often used with caution in poultry diets, as fiber is traditionally considered an antinutritional factor for non-ruminants (Souza et al. 2019). However, studies investigating different sources and levels of fiber inclusion have demonstrated beneficial effects on nutrient metabolism and digestibility (González-Alvarado et al. 2010; Jiménez-Moreno et al. 2019), highlighting the potential of some agro-industrial by-products, rich in fiber, for poultry diet formulation.

Gut health in animals is closely linked to diet composition, particularly through its influence on the proliferation of beneficial and pathogenic bacteria. According to Montagne et al. (2003), dietary fiber is considered a key component in this regard. When fermented by intestinal bacteria, soluble fiber produces short-chain fatty acids (SCFAs), primarily acetate, propionate, and butyrate, which lower intestinal pH and can modulate the growth of certain pathogenic bacteria, including *Salmonella*, *Clostridium*, *Escherichia coli*,* and Clostridium difficile*.

Moderate inclusion of dietary fiber also benefits intestinal morphology, enhancing nutrient digestion and absorption (Jiang et al. 2024), and serves as an immunostimulant by increasing T and B lymphocyte production (Hussein et al. 2023). Thus, carefully balanced fiber levels in diets can achieve these effects without the need for additional immunostimulatory additives (Montagne et al. 2003; Goulart et al. 2016).

In slow-growing broiler breeds, small-scale producers often empirically include wheat bran in diets to partially replace corn and soybean meal as a cost-effective strategy. Wheat bran increases intestinal viscosity due to its high soluble non-starch polysaccharide (NSP) content, particularly arabinoxylans, which can reduce the digestibility of carbohydrates, proteins, and lipids, increase endogenous protein loss, and cause enterocyte shedding (Kim et al. 2022). Nevertheless, moderate levels of wheat bran can improve antioxidant status, gizzard development, digestive enzyme activity, and intestinal morphology in birds (Ali, 2008; Shang et al. 2020).

Guava residue is another alternative feed gaining interest among slow-growing poultry producers. This by-product has high crude fiber content (~ 61%), resulting in low metabolizable energy for birds (~ 1401 kcal/kg) (Guimarães et al., 2007). Studies have reported that inclusion of up to 12–15% guava residue in diets of fast-growing (conventional) broilers and slow-growing broilers, respectively, did not negatively affect performance (Lira et al. 2009; Vieira et al. 2023). In contrast, Ogega et al. (2022) observed reduced weight gain in broilers fed 7.5% guava residue (46.4% crude fiber, 5.4% CP), recommending inclusion of this by-product at levels no higher than 5%.

The hypothesis of this study was that moderate NDF inclusion from alternative sources, particularly guava residue, would not impair growth performance, could improve meat sensory attributes, and might exert a modulatory effect on the cecal microbiota of slow-growing broilers.

Therefore, this study aimed to evaluate the effects of increasing neutral detergent fiber (NDF) levels from two sources, wheat bran or guava residue, on performance, meat quality, and intestinal microbiota of slow-growing broiler chickens.

## Materials and methods

### Location and facilities

The experiment was conducted at the Experimental Farm of Vale do Acaraú State University (UVA), in the Experimental Warehouse for Broiler Chickens, located in Sobral, Ceará, Brazil (3°36′ S, 40°18′ W; altitude 56 m). The region has an average annual temperature of 27.6 °C, relative humidity of 68.7%, and an average annual rainfall of 808 mm, mostly concentrated in the first five months of the year. The predominant climate is classified as BSh (dry, semi-arid, low latitude and altitude) according to the Köppen system.

Birds were housed in a masonry poultry house and allocated to floor pens (1.0 × 1.5 m) with wire-mesh partitions and concrete floors covered with wood shavings litter. Each pen was equipped with a pendulum drinker and a tubular feeder. Artificial heating was provided by infrared lamps positioned approximately 20 cm above the birds. Curtains and lamps were adjusted according to bird behavior to maintain optimal environmental conditions.

### Animals and experimental design

The study was conducted in two phases: the initial phase, from 1 to 28 days of age (Experiment 1), and the growth/final phase, from 29 to 70 days of age (Experiment 2). All procedures were carried out in accordance with the project approved by the Ethics Committee for the Use of Animals (CEUA) of Vale do Acaraú State University (UVA), under protocol No. 002.03.024.UVA.504.03.

A total of 400 one-day-old slow-growing Naked Neck chicks were purchased to select birds for the experiments. In both experiments, birds were assigned to a 1 + (2 × 2) factorial design, resulting in five treatments with five replicates each. All chicks were vaccinated against Marek’s disease at the hatchery and immunized against Newcastle disease via the ocular route at 7 days of age.

In Experiment 1, eight unsexed birds per replicate were assigned to five treatments, totaling 200 birds. Birds were individually weighed and distributed in a completely randomized design to ensure homogeneity of initial body weight across treatments (36.74 g ± 0.40 g), occupying 25 pens. The remaining 200 chicks not used in the first experiment were raised in the available pens and were fed the same diet, formulated with corn and soybean meal to meet the nutritional requirements for slow-growing broilers from 1 to 28 days of age, according to Figueiredo et al. (2022), and were subsequently used in Experiment 2.

At the end of Experiment 1 (28 days), the first experiment was concluded, and the second experiment was initiated, covering 29 to 70 days. Birds not used in the first experiment were employed to avoid carryover effects. Each bird was individually weighed and sexed, and the experiment was arranged in a randomized block design, with sex as the blocking factor. In this phase, six birds per replicate were assigned to five treatments, totaling 150 birds. The initial body weight of the birds in Block 1 (males) was 817.2 g ± 10.5 g, while the birds in Block 2 (females) had an initial body weight of 709.6 g ± 11.1 g.

### Experimental treatments and diets

The experimental treatments consisted of a control diet (CD) with fiber content (11.47% of neutral detergent fiber - NDF), formulated from corn and soybean meal, and four diets supplemented with NDF in a 2 × 2 factorial design: 3% or 6% NDF from two fiber sources, wheat bran (WB) or guava residue (GR), resulting in the following treatments: CD, WB3%, WB6%, GR3%, and GR6%.

Diets (Table 1) were formulated to meet the nutritional requirements of slow-growing broilers (Figueiredo et al., 2022), using ingredient composition data from Rostagno et al. (2017). The guava residue was obtained as a by-product from the Sabor da Serra Pulp Factory (Meruoca, Ceará, Brazil), consisting mainly of seeds and a small proportion of fruit peel remaining after industrial pulp extraction. After collection, the residue was frozen until transport to the Experimental Farm. It was then sun-dried on plastic tarps for four days, with turning twice daily and covering at night. After drying, the material was ground using a 4-mm screen and stored in bags on wooden pallets prior to diet mixing. Bromatological analysis of the guava residue was conducted at the Animal Nutrition Laboratory (LANUT) of UVA, yielding the following composition on an as-fed basis: dry matter (DM) 95.8%, crude protein (CP) 10.9%, mineral matter (MM) 3.1%, ether extract (EE) 10%, crude fiber (CF) 37.0%, neutral detergent fiber (NDF) 52.52%, and acid detergent fiber (ADF) 32.17%. Calcium and phosphorus contents were obtained from Lousada Júnior et al. (2006), and metabolizable energy values were taken from Silva et al. (2009).

**Table 1:** Percentual nutritional composition of the experimental diets ^1^

Ingredients	Experiment 1 (1 to 28 days)					Experiment 2 (29 to 70 days)				
		NDF Source					NDF Source			
	Control	Wheat Bran		Guava Residue		Control	Wheat Bran		Guava Residue	
		NDF content		NDF content			NDF content		NDF content	
		+ 3%	+ 6%	+ 3%	+ 6%		+ 3%	+ 6%	+ 3%	+ 6%
Corn grain	59.757	56.706	44.375	57.958	52.427	66.623	60.340	47.518	64.227	55.938
Soybean meal 46%	32.831	30.289	28.512	32.023	31.718	27.377	25.101	23.365	26.649	26.716
Wheat bran	-	9.138	20.628	-	-	-	9.958	21.572	-	-
Guava residue​	-	-	-	5.176	10.911	-	-	-	5.267	11.410
Soybean oil	0.000	0.000	2.857	0.000	1.190	0.000	0.995	4.004	0.190	2.260
Dicalcium phosphate	1.999	1.872	1.725	2.004	2.016	1.854	1.720	1.572	1.861	1.877
Limestone	1.027	1.100	1.178	1.025	1.017	1.162	1.237	1.314	1.159	1.146
Common salt	0.365	0.367	0.370	0.368	0.371	0.296	0.298	0.302	0.298	0.302
DL-Methionine	0.063	0.053	0.055	0.054	0.050	0.056	0.051	0.053	0.049	0.048
Premix ^1^	0.300	0.300	0.300	0.300	0.300	0.300	0.300	0.300	0.300	0.300
Inert	3.659	0.000	0.000	1.092	0.000	2.331	0.000	0.000	0.000	0.000
Total	100.0	100.0	100.0	100.0	100.0	100.0	100.0	100.0	100.0	100.0
ME (kcal/kg)	2800	2800	2800	2800	2800	2900	2900	2900	2900	2900
Crude protein (%)	20.0	20.0	20.0	20.0	20.0	18.0	18.0	18.0	18.0	18.0
Calcium (%)	1.00	1.00	1.00	1.00	1.00	1.00	1.00	1.00	1.00	1.00
Available Phosphorus ^2^ (%)	0.47	0.47	0.47	0.47	0.47	0.43	0.43	0.43	0.43	0.43
Sodium (%)	0.18	0.18	0.18	0.18	0.18	0.15	0.15	0.15	0.15	0.15
Met + cis ^3^ (%)	0.70	0.70	0.70	0.70	0.70	0.65	0.65	0.65	0.65	0.65
Methionine (%)	0.38	0.37	0.36	0.37	0.38	0.35	0.34	0.35	0.34	0.34
Lysine (%)	1.06	1.06	1.03	1.04	1.06	0.93	0.91	0.93	0.91	0.91
Threonine (%)	0.78	0.77	0.78	0.77	0.78	0.70	0.70	0.70	0.70	0.70
CF (%)	2.97	3.59	4.30	5.29	7.94	2.77	3.42	4.14	5.17	7.94
NDF (%)	11.47	14.47	17.47	14.47	17.47	11.48	14.48	17.48	14.48	17.48
ADF (%)	4.60	5.33	6.06	7.44	10.52	4.41	5.14	4.41	7.29	10.54

Vitamin-mineral supplement: guaranteed levels per kg of product = 3,333.33 mg of copper; 16.66 mg of iron; 333.33 mg of iodine; 20 g of manganese; 116.66 mg of selenium; 23.33 g of zinc; 4,000,000 IU of vit THE; 1,166,667 IU of vit D3; 13,333.33 vit E; 1,000 mg of vit K3; 833.33 mg of vit B1; 2,333.33 of vit B2; 1,333.33 mg of vit B6; 10,000 micrograms of vit B12; 666.66 folic acid; 16.66 g of nicotinic acid; 5,000 mg of pantothenate; 50 mg of biotin and 104 g of choline; ^2^ Available phosphorus; ^3^ Methionine + cysteine

**Table 2:** Performance of slow-growing chickens in the initial (Experiment 1) and growth/finishing (Experiment 2) phases, subjected to different sources and levels of NDF

Diets	Experiment 1 (1 to 28 days)				Experiment 2 (29 to 70 days)			
	BW (g/bird)	FI (g/bird)	WG (g/bird)	FCR (g/g)	BW (g/bird)	FI (g/bird)	WG (g/bird)	FCR (g/g)
Control	680.4	1218.9	643.6	1.898	2819.0	5745.2	2046.2	2.828
WB3%	490.4*	1101.8	453.6*	2.517*	2904.8	5314.4	2113.3	2.628
WB6%	617.3	1345.9	580.6	2.321*	2717.2	5643.4	1941.8	2.929
GR3%	649.5	1185.9	612.7	1.941	2781.8	5566.6	2006.4	2.794
GR6%	574.0*	1302.2	537.3*	2.425*	2953.0	5869.5	2160.9	2.743
SEM	17.2	25.8	17.2	0.068	18.1	65.0	55.2	0.048
WB	553.9	1223.8	517.1	2.419	2811.0	5478.9	2027.6	2.779
GR	611.7	1244.0	575.0	2.183	2867.4	5718.1	2083.7	2.769
3%	569.9	1143.9 b	533.2	2.229	2843.3	5440.5 b	2059.85	2.711
6%	595.7	1324.0 a	558.9	2.373	2835.1	5756.4 a	2051.35	2.836
SEM	14.1	25.2	14.1	0.061	18.5	75.61	18.5	0.075
Effect								
Source	0.0673	0.7043	0.0679	0.0828	0.1708	0.0900	0.1836	0.8740
Content	0.3945	0.0036	0.3952	0.2750	0.8369	0.0309	0.8354	0.0741
Source x Content	0.0036	0.2401	0.0037	0.0173	0.0642	0.3017	0.0719	0.0800

*Means followed by an asterisk differ from the control diet, according to the Dunnett’s test, at 5% probability (*n* = 5); ^a, b^ Means followed by different uppercase letters in the row differ statistically from each other, according to the Tukey’s test, at 5% probability; BW = Body weight; FI = Feed intake; WG = Weight gain; FCR = Feed conversion ratio; WB3% = diet with + 3% NDF and wheat bran; WB6% = diet with + 6% NDF and wheat bran; GR3% = diet with + 3% NDF and guava residue; GR6% = diet with + 6% NDF and guava residue; NDF = Neutral detergent fiber; SEM = Standard error of the mean

To achieve the desired NDF levels, the CD was first formulated, and 3% or 6% additional NDF was incorporated using WB or GR. Energy levels were adjusted with inert material or soybean oil to maintain isoenergetic diets. All diets were iso-protein and contained equal levels of calcium, available phosphorus, sodium, methionine + cysteine, and lysine (Table 1).

Throughout the experiment, birds had ad libitum access to feed and chlorinated water. Drinkers were cleaned daily, and feeders were refilled as needed to prevent feed shortages.

### Evaluation of performance, cecal microbiota, and carcass traits

Birds and feed were weighed at the beginning and end of each experimental phase to calculate feed intake, body weight, weight gain, and feed conversion ratio. Body weight gain was determined as the difference between the final and initial body weights of the birds, and feed conversion ratio was calculated as the ratio of total feed intake to body weight gain. In cases of mortality, leftover feed and dead birds were weighed to make the necessary adjustments.

Productive performance and cecal microbiota evaluation were conducted at the end of each experiment (28 and 70 days of age), while carcass and meat cut yields, as well as sensory analysis, were performed only in the second experiment (70 days of age). For sampling, five birds per treatment (one per replicate), with body weights within 10% of the replicate mean, were randomly selected. After an 8-hour fasting period, birds were weighed and euthanized by cervical dislocation (CONCEA, Normative Resolution No. 37, February 15, 2018). In the first experiment, only organ dissection was performed, whereas in the second experiment, birds were bled, scalded, and plucked. The abdomen was incised with a sterile scalpel to remove the ceca, which were immediately weighed and placed in a labeled universal container. Sterile water was added to prevent sample desiccation before transfer to the Microbiology Laboratory at Vale do Acaraú State University. New scalpels and gloves were used for each sample to prevent cross-contamination.

After cecum collection, the remaining viscera, head, and feet were removed, and the carcass was weighed to calculate carcass yield relative to slaughter weight. Yields of meat cuts (breast, thigh, drumstick, and wing) were expressed relative to carcass weight. The relative weights of edible viscera (heart, liver, and gizzard), abdominal fat, small intestine, and lymphoid organs (spleen and Bursa of Fabricius) were expressed relative to slaughter weight.

### Microbiological analysis

For microbiological analyses, the Most Probable Number (MPN) of Total Coliforms (TC) and Thermotolerant Coliforms (TTC) was determined using the multiple-tube fermentation method (Blodgett 2003). The procedure consisted of three steps: the presumptive test, the confirmatory test for total and thermotolerant coliforms, and the biochemical test (Feng et al. 2020).

For the presumptive analysis, 25 g of cecal content per treatment were weighed and homogenized with 225 mL of sterile saline solution (0.85%), resulting in a 10⁻¹ dilution. One milliliter of this solution was sequentially diluted in 9 mL of saline to obtain 10⁻² and 10⁻³ dilutions. From each dilution, 1 mL was inoculated into 10 mL of Lactose Broth in sterilized test tubes, following the same procedure for 10⁻² and 10⁻³ dilutions. The tubes were incubated at 36 °C for 48 h. Three dilutions were analyzed for each of the 15 samples, corresponding to triplicates of each of the five treatments (Feng et al. 2020).

For the determination of Total and Thermotolerant Coliforms, aliquots from positive Lactose Broth tubes were transferred to Brilliant Green Bile Broth and incubated at 35 °C for 48 h to quantify total coliforms. Subsequently, using a chrome-nickel loop, aliquots from the positive tubes were inoculated into *Escherichia coli* broth and incubated in a water bath at 45 °C for 48 h. Samples that exhibited turbidity in the medium were considered positive.

For the quantification and isolation of *Escherichia coli*, aliquots were taken from positive tubes of *E. coli* broth and inoculated onto plates containing Eosin Methylene Blue (EMB) agar using a chrome-nickel loop. The plates were then incubated at 36 °C for 24 h (Koneman and Win 2008). Samples showing growth of isolated or clustered colonies were considered positive.

The biochemical test, also known as the IMViC test, comprises a series of four assays used to characterize microorganisms of the *Enterobacteriaceae* family (Koneman and Win 2008). The reagents employed were indole, methyl red, Voges-Proskauer, and citrate. In the citrate medium, a color change to blue was interpreted as positive, while maintenance of the green color was considered negative. For the other media, samples displaying turbidity along with the characteristic color reaction of the microorganism were classified as positive.

To quantify mesophilic aerobic microorganisms, the *pour plate* technique was employed. From each dilution, 1 mL was transferred to 9 mL of saline solution. An aliquot of the homogenized solution, corresponding to each dilution, was then dispensed into Petri dishes and overlaid with 15 mL of standard counting agar. The inoculum was spread using a chrome-nickel loop in a figure-of-eight motion to ensure uniform distribution. After solidification of the medium, the plates were incubated at 35 °C for 24 h (Silva et al. 2001).

Colony counts were performed on Petri dishes containing thin-layer potato agar using a colony counter and a magnifying glass. Only oval-shaped clusters were considered. Plates with more than 250 colonies were classified as uncountable.

### Sensory analysis

For the sensory analysis of chicken meat, five breasts from each treatment were collected, labeled, and frozen. Prior to evaluation, the samples were thawed at 5 °C for 24 h. The sensory panel consisted of 61 untrained assessors from the UVA academic community, including students, professors, and staff, all in good health and without dietary restrictions related to poultry consumption.

Chicken breasts were cut into 2 cm cubes (approximately 20 g) and cooked on a grill. The samples were placed in an electric oven preheated to 170 °C and cooked until reaching an internal temperature of 72 °C ± 1 °C, monitored with a skewer thermometer. The cooking process lasted approximately 12 min, with six minutes on each side. After cooking, the samples were held in a water bath at 55 °C until serving. Finally, they were cut into 1.5 × 1.5 cm pieces and served immediately to the panelists in individual booths at the Agricultural Products Technology Laboratory (TPA), UVA.

The acceptance test was conducted using the nine-point hedonic scale proposed by Dutcosky (2011), ranging from “I liked it extremely (9)” to “I disliked it extremely (1),” to evaluate the attributes of appearance, color, aroma, flavor, tenderness, and juiciness. In addition, a preference test was performed using a five-point scale, ranging from “I liked it the least (1)” to “I liked it the most (5).” Each sample was coded with a random three-digit number, and panelists were instructed to taste the samples in sequence. To cleanse their palate between samples, water and/or saltine crackers were provided in individual booths.

### Water-holding capacity and cooking weight loss

For the determination of water-holding capacity (WHC) and cooking weight loss (CWL), five samples per treatment were used, corresponding to one sample per experimental replicate. These samples were prepared from the previously collected, identified, and stored chicken breasts.

Water-holding capacity (WHC) was determined following the method of Hamm (1961). Water loss was assessed by applying pressure to muscle tissue. Small portions of chicken breast fillet (≈ 0.5 g) were placed between two filter papers and positioned between glass plates. A 5 kg weight was applied for 5 min. After pressure application, the samples were reweighed, and water loss was calculated by difference. Results were expressed as the percentage of water retained (initial weight minus exuded water) relative to the initial sample weight.

Cooking weight loss (CWL) was determined by weighing 2 cm cubes of chicken breast before and after cooking on an electric grill, using an analytical scale. Samples were cooked until reaching an internal temperature of 72 °C ± 1 °C. CWL was calculated using the formula: (100 – ((final weight/initial weight) x 100). The average CWL was then calculated for each treatment.

### Statistical analysis

The data were subjected to the Shapiro–Wilk normality test to check for a normally distributed population. Variables that met the assumption of normal distribution were analyzed using factorial ANOVA, followed by Dunnett’s test, comparing diets with increasing levels of NDF to the control diet (CD). Sensory attributes were evaluated using the nonparametric Kruskal–Wallis test. When a significant effect was detected, multiple comparisons among treatments were performed using the Mann–Whitney test with Bonferroni correction to adjust the significance level. A 5% significance level was adopted for all analyses. For microbiological data, the formation of cecal sample pools per treatment (*n* = 1 per group) precluded inferential statistical analysis. Therefore, microbiological results are presented descriptively, based on the comparison of observed absolute values.

## Results

### Performance, carcass yield, meat cut yields, and organ biometrics

#### Experiment 1 (1 to 28 days)

Compared to the control diet (CD), body weight (BW) and weight gain (WG) were significantly lower in birds fed the WB3% (*P* < 0.05) and GR6% (*P* < 0.05) diets. No significant differences in BW and WG were observed among the other treatments (Table 2). Feed conversion ratio (FCR) was significantly worse in birds fed the WB3% (*P* < 0.05), WB6% (*P* < 0.05), and GR6% (*P* < 0.05) diets compared to the CD. Only the GR3% diet resulted in an FCR similar to the CD (*P* > 0.05). Feed intake (FI) did not vary significantly among the experimental treatments (*P* > 0.05).

Regarding the factorial analysis, the source of NDF (SNDF) had no significant effect on any performance trait (*P* > 0.05). However, the level of NDF inclusion (LOI) significantly influenced FI (*P* = 0.0036), with birds fed 6% NDF having higher intake than those fed 3% NDF. The LOI had no significant effect on BW (*P* = 0.3945), WG (*P* = 0.3952), or FCR (*P* = 0.2750). A significant interaction effect between SNDF and LOI was identified for BW (*P* = 0.0036), WG (*P* = 0.0037), and FCR (*P* = 0.0173).

The breakdown of this interaction (Table 3) showed that when wheat bran was used as the fiber source, performance (BW, WG, FCR) was significantly better with 6% NDF compared to 3% NDF (*P* < 0.05). In contrast, no differences were observed between the NDF levels when guava residue was used as the fiber source (*P* > 0.05). Furthermore, with the addition of 6% NDF, no differences were found between the two fiber sources. However, with 3% NDF, performance was significantly impaired when wheat bran was used compared to guava residue.

**Table 3:** The interaction effect of source of NDF (SNDF) and level of inclusion (LOI) on weight body mass, weight gain and feed conversion ratio of slow-growing chickens, in Experiment 1

SNDF	Body weight (g/bird)		Weight gain (g/bird)		Feed conversion ratio (g/g)	
	+ 3% NDF	+ 6% NDF	+ 3% NDF	+ 6% NDF	+ 3% NDF	+ 6% NDF
Wheat bran	490.4Bb	617.3Aa	453.6Bb	580.0Aa	2.517Ba	2.321Aa
Guava residue	649.6Aa	574.0Aa	612.7Aa	537.3Aa	1.941Aa	2.224Aa
SEM	18.5		18.5		0.075	

^A, B^ Means followed by different uppercase letters in the row differ statistically from each other, according to the Tukey’s test, at 5% of probability (*n* = 5); ^a, b^ Means followed by different lowercase letters in the column differ statistically from each other, according to the Tukey’s test, the 5% of probability; NDF = Neutral detergent fiber; SEM = Standard error of the mean

#### Experiment 2 (29 to 70 days)

No significant differences in BW, WG, FCR, or FI were observed between birds fed the CD and those receiving any of the NDF-supplemented diets (*P* > 0.05) (Table 2).

The factorial analysis revealed no significant interaction between SNDF and LOI for BW (*P* = 0.0642), FI (*P* = 0.3017), WG (*P* = 0.0719), or FCR (*P* = 0.0800). Similarly, the SNDF had no significant effect on BW (*P* = 0.1708), FI (*P* = 0.0900), WG (*P* = 0.1836), or FCR (*P* = 0.8740). The LOI significantly influenced FI (*P* = 0.0309), being higher in birds fed 6% NDF, but had no significant effect on BW (*P* = 0.8369), WG (*P* = 0.8354), or FCR (*P* = 0.0741).

#### Carcass traits and organ biometrics

Compared to the CD, only the relative gizzard weight was significantly higher in birds fed the GR6% diet (*P* < 0.05). No other significant effects of NDF-supplemented diets were observed on carcass yield, meat cut yields, or the relative weight of the remaining organs and abdominal fat (*P* > 0.05) (Table 4).

**Table 4:** Yield (%) of carcass and meat cuts, and relative weight (%) of viscera, lymphoid organs and abdominal fat of slow-growing chickens aged 29 to 70 days (*n* = 5), fed diets with different sources and levels of NDF

Diets	Carcass	Breast	Thigh	Drumstick	Wing	Heart	Liver	Gizzard	Spleen	Bursa	Fat
CD	74.9	30.0	15.6	15.8	11.6	0.42	1.50	1.59	0.12	0.18	1.44
WB3%	75.9	29.7	15.6	16.5	11.6	0.40	1.29	1.75	0.11	0.20	1.86
WB6%	75.6	30.1	14.6	15.8	11.7	0.39	1.34	1.68	0.12	0.24	1.79
GR3%	75.8	30.9	15.2	16.1	11.7	0.36	1.43	1.83	0.12	0.19	1.47
GR6%	76.0	29.0	15.4	16.2	11.6	0.38	1.64	2.10*	0.14	0.21	1.46
SEM	0.28	0.48	0.24	0.15	0.10	0.01	0.05	0.06	0.01	0.01	0.21
WB	75.8	29.9	15.1	16.2	11.7	0.39	1.32	1.71	0.11	0.22	1.82
GR	75.9	29.9	15.3	16.2	11.6	0.37	1.54	1.96	0.13	0.20	1.46
3%	75.9	30.3	15.4	16.3	11.7	0.38	1.36	1.79	0.11	0.19	1.67
6%	75.8	29.5	15.0	16.0	11.6	0.38	1.49	1.89	0.13	0.22	1.62
SEM	0.22	0.48	0.24	0.17	0.09	0.01	0.05	0.06	0.01	0.01	0.013
Effect	……………………………………………………….Probability…………………………………………………………….										
Source	0.8308	0.9617	0.7056	0.9961	0.9680	0.1543	0.0293	0.0924	0.3838	0.5338	0.1927
Content	0.8587	0.3029	0.4201	0.3525	0.9299	0.6318	0.1680	0.4708	0.4044	0.3386	0.8701
S x C	0.7352	0.3005	0.4040	0.4080	0.4088	0.3767	0.2401	0.2251	0.4269	0.8025	0.9049

*Means followed by an asterisk differ from the control diet, according to the Dunnett’s test, at 5% probability (*n* = 5); WB3% = diet with + 3% NDF and wheat bran; WB6% = diet with + 6% NDF and wheat bran; GR3% = diet with + 3% NDF and guava residue; GR6% = diet with + 6% NDF and guava residue; NDF = Neutral detergent fiber; SEM = Standard error of the mean

The two-way ANOVA showed no significant interaction (SNDF x LOI) for any of these variables (all *p* > 0.22). Furthermore, there was no isolated effect of the SNDF on carcass yield (*p* = 0.8308), yields of breast (*p* = 0.9617), thigh (*p* = 0.7056), drumstick (*p* = 0.9961), wings (*p* = 0.9680), or on the relative weight of heart (*p* = 0.1543), gizzard (*p* = 0.0924), spleen (*p* = 0.3838), bursa (*p* = 0.5338), and abdominal fat (*p* = 0.1927). The LOI also did not influence these carcass yields or the relative weights of heart (*p* = 0.6318), liver (*p* = 0.1680), gizzard (*p* = 0.4708), spleen (*p* = 0.4044), bursa (*p* = 0.3386), and abdominal fat (*p* = 0.8701). The only exception was the relative liver weight, which was significantly influenced by the SNDF (*p* = 0.0293), with birds fed guava residue-based diets presenting heavier livers than those fed wheat bran-based diets.

#### Cecal microbiota

Due to limited sample quantity, microbiological analyses were performed using pooled samples per treatment. Consequently, the results represent observed values and numerical trends and are not subject to formal inferential statistical analysis. Based on the composite sample values, no numerical variation was observed for mesophilic aerobic bacteria among treatments in either experimental phase, with all groups showing the same count of 2.5 × 10⁵ CFU/g. Regarding thermotolerant coliforms, in the initial phase (28 days) treatments with wheat bran (WB3% and WB6%) and with 3% guava residue (GR3%) showed numerically lower counts (ranging from 7.0 × 10¹ to 3.3 × 10¹ CFU/g) compared to the control diet (CD) and the GR6% treatment (both with 1.6 × 10³ CFU/g). In the growth/finishing phase (70 days), all NDF-supplemented treatments showed numerically lower counts than the control diet. For *Escherichia coli* prevalence, the observed values varied among treatments. In the initial phase, the lowest prevalence was observed in the WB6% treatment (9.5%) and the highest in the GR6% treatment (33.3%). In the finishing phase, the trend of lower prevalence in the WB6% treatment (8.0%) was maintained, while the GR6% treatment showed a value of 24.0% (Table 5).

**Table 5:** Count of thermotolerant coliforms, mesophilic aerobic bacteria and prevalence of *Escherichia coli* in the cecum region of slow-growing chickens, subjected to different sources and levels of NDF

Diets	Experiment 1 - Initial phase (28 days)				Experiment 2 - Growth/Finishing Phase (70 days)		
	Thermotolerant Coliforms (CFU/g)	Mesophilic Aerobes (CFU/g)	Prevalence of Escherichia coli (%)		Thermotolerant Coliforms (CFU/g)	Mesophilic Aerobes (CFU/g)	Prevalence of Escherichia coli (%)
CD	1.6 × 10	2.5 × 10	19.0		9.0 × 10	2.5 × 10	36.0
WB3%	7.0 × 10	2.5 × 10	23.8		9.0 × 10	2.5 × 10	16.0
WB6%	9.0 × 10	2.5 × 10	9.5		3.3 × 10	2.5 × 10^5^	8.0
GR3%	3.3 × 10	2.5 × 10^5^	14.3		3.4 × 10	2.5 × 10^5^	16.0
GR6%	1.6 × 10^3^	2.5 × 10^5^	33.3		3.4 × 10	2.5 × 10^5^	24.0

WB3% = diet with + 3% NDF and wheat bran; WB6% = diet with + 6% NDF and wheat bran; GR3% = diet with + 3% NDF and guava residue; GR6% = diet with + 6% NDF and guava residue; NDF = Neutral detergent fiber; SEM = Standard error of the mean

### Sensory and physical quality of chicken breast and consumer preference

The appearance and color of broiler breast meat were positively influenced by increasing NDF levels from both sources, with the effect being more pronounced when 6% NDF from guava residue was included. For juiciness, the diet with 6% NDF from guava residue resulted in higher scores compared with the diet containing 6% NDF from wheat bran, while all other diets showed similar values. No dietary effects were observed on aroma, flavor, tenderness, or water-holding capacity. Cooking loss was lower for the GR3% diet compared with the GR6% and WB3% diets, with no differences from the other treatments (Table 6).

**Table 6:** Sensory and physical characteristics of meat from slow-growing chickens at 70 days of age, subjected to diets with different sources and levels of NDF

Diets	Appearance	Color	Aroma	Flavor	Tenderness	Juiciness	WHC	CWL
CD	6.18 c	5.88 c	6.45	6.37	7.14	6.67 ab	64.49	27.31 ab
WB3%	7.19 b	7.06 b	6.75	6.90	7.16	6.96 ab	66.42	28.56 a
WB6%	7.26 b	6.98 b	6.96	6.27	7.16	6.45 b	61.44	26.67 ab
GR3%	6.98 b	6.93 b	6.65	6.70	7.11	6.68 ab	62.15	23.85 b
GR6%	8.21 a	8.03 a	7.18	7.04	7.54	7.37 a	64.31	27.82 a
p-value (KW)	0.0001	0.0001	0.1685	0.0623	0.5026	0.0217	0.2998	0.0148
SEM	0.32	0.34	0.34	0.34	0.30	0.32	0.61	0.47

^a, b,c^ Means followed by different letters in the column are different from each other according to the Mann–Whitney test with Bonferroni adjustment at the 5% significance level; SEM: Standard Error of the Mean. at 5% probability (*n* = 5); WHC = Water-holding capacity; CWL = Cooking weight loss; CD = Control diet; WB3% = diet with + 3% NDF and wheat bran; WB6% = diet with + 6% NDF and wheat bran; GR3% = diet with + 3% NDF and guava residue; GR6% = diet with + 6% NDF and guava residue; NDF = Neutral detergent fiber; KW = Kruskal-Wallis test; SEM = Standard error of the mean

Regarding preference, untrained panelists preferred the breast meat from broilers fed the GR6% diet (32.8%), followed by WB3% (29.5%), while the least appreciated was from the WB6% diet (6.6%). The breast meat from broilers fed the control diet (CD) and the GR3% diet was preferred by 16.5% and 14.8% of consumers, respectively.

## Discussion

The physiological effects of dietary fiber in broilers depend on multiple factors, including fiber source, inclusion level, and bird age (Berrocoso et al. 2020). The divergent results between the initial and growth phases in this study align with the established concept of gastrointestinal tract maturation. Young birds possess an immature digestive system with limited capacity for fiber fermentation (Bautil et al. 2019).

In Experiment 1, the impaired body weight (BW) and weight gain (WG) in birds fed the WB3% and GR6% diets, and the worse feed conversion ratio (FCR) in birds fed WB3%, WB6%, and GR6% diets compared to the control (CD), can be primarily attributed to the physical and antinutritive effects of insoluble fibers in an underdeveloped gut. The high fiber content, particularly from the insoluble fraction in wheat bran and the increased level in guava residue, likely increased digesta viscosity and passage rate, reducing the contact time between nutrients and digestive enzymes, thus impairing nutrient digestibility and utilization (Jha et al. 2019; Singh and Kim 2021). The dose-dependent nature of this response is further illustrated when comparing our findings with those of Oliveira et al. (2018). These authors evaluated much lower inclusion levels of guava by-product (0.5% to 1.5%) in the starter phase and reported no performance depression, with even a linear improvement in weight gain at 7 days. In contrast, our study demonstrates that when guava residue inclusion reaches approximately 10.9% (equivalent to 6% additional NDF), it negatively affects BW, WG, and FCR in young birds. However, the GR3% diet (~ 5.2% guava residue) supported performance comparable to the control diet, indicating that this intermediate level represents a tolerable threshold for slow-growing chicks in the initial phase.

Thus, while very low inclusions of guava residue may offer nutritional or bioactive benefits (Oliveira et al. 2018), excessive fiber load from higher inclusions overwhelms the immature digestive system. The GR3% level appears to balance the provision of fermentable soluble fiber (e.g., pectin) with the physical limitations of the underdeveloped gastrointestinal tract, without severely compromising gut function or nutrient utilization.

Conversely, in Experiment 2, none of the performance traits (BW, WG, FI, FCR) differed between the CD and the NDF-supplemented diets. This can be explained by the full development of the gastrointestinal tract and its microbial ecosystem in older birds. A mature gut microbiota enhances the fermentation of dietary fibers into short-chain fatty acids (SCFAs), which serve as an additional energy source and promote intestinal health (Shang et al. 2020). This compensatory mechanism likely allowed birds to adapt to the higher fiber content, neutralizing any negative impact on performance. Furthermore, the formulated diets were isoenergetic and isoprotein, ensuring that nutritional requirements were met despite the dilutive effect of fiber, which explains the maintained performance.

Notably, the source of NDF (wheat bran vs. guava residue) had no isolated significant effect on any performance trait in either phase. This indicates that, within the levels tested, the physical and chemical properties of both fiber sources elicited similar overall physiological responses concerning nutrient utilization for growth.

Regarding carcass and organ traits, the lack of significant differences in yields and most organ weights (except liver) among treatments is consistent with the isoenergetic and isoprotein formulation of the diets, as these factors are primary determinants of carcass composition. The similar relative gizzard weights across treatments were unexpected, as insoluble fibers often stimulate gizzard development (Jha et al., 2021). This suggests that the inclusion levels and/or fiber characteristics in this study were insufficient to induce significant muscular hypertrophy in this organ.

The only significant finding for organs was the higher relative liver weight in birds fed guava residue-based diets compared to wheat bran-based diets. This could be related to the liver’s role in metabolizing bioactive compounds (such as carotenoids) present in guava residue, potentially increasing metabolic activity and organ size. However, the literature presents divergent findings regarding liver weight in response to dietary guava by-products. Vieira et al. (2023) observed a quadratic effect on liver yield in slow-growing broilers, with higher relative weights at 5% and 10% dehydrated guava by-product, followed by a reduction at 15% inclusion. In contrast, Melo et al. (2026) found no significant differences in liver weight across inclusion levels up to 20% guava residue in isoenergetic diets.

An opposite trend was reported by Lana et al. (2020), who observed a reduction in liver weight of European quails fed increasing levels of guava residue. It is important to highlight that, in their study, the inclusion of guava residue progressively reduced the dietary energy content, requiring the addition of increasing amounts of soybean oil to maintain isoenergetic conditions. The authors suggested that this higher dietary oil content may have suppressed hepatic lipogenesis, leading to reduced liver mass. Therefore, the reduction in liver weight was likely not a direct effect of guava residue per se, but rather a consequence of the metabolic adaptation to a higher lipid intake.

These discrepancies indicate that liver response to guava residue is influenced by multiple factors, including inclusion level, bird age and genotype, diet formulation strategy (isoenergetic vs. non-corrected), and the balance between bioactive compounds and energy sources such as soybean oil.

For meat quality, the significantly higher appearance and color scores for the GR6% diet are likely attributed to the deposition of carotenoid pigments (β-carotene and lycopene) naturally present in guava residue into the muscle tissue (Baéza et al. 2022). This finding is strongly supported by recent studies. Vieira et al. (2023) reported a linear increasing effect on breast and thigh skin coloration with increasing dietary guava by-product, and Melo et al. (2026) observed that inclusion levels above 10% GR significantly improved meat color scores. Oliveira et al. (2018), although working with younger birds (21 days), found no significant differences in colorimetry parameters, possibly due to the shorter feeding period or lower inclusion levels (up to 1.5%). The authors did, however, report a quadratic reduction in lipid oxidation (TBARS) in thigh meat, with the lowest value estimated at 0.72% inclusion, demonstrating the antioxidant potential of guava phenolic compounds. This enhanced visual appeal directly contributed to this treatment receiving the highest consumer preference.

The higher juiciness score for the GR6% diet compared to the WB6% diet suggests a difference in the meat’s water-holding capacity or lipid content. It is plausible that the dietary composition of the GR6% diet, potentially influencing the muscle’s fatty acid profile or intramuscular fat content, resulted in a juicier sensory perception (Baéza et al. 2022). Melo et al. (2026) did not detect significant differences in juiciness, tenderness, or flavor, but reported significant improvements in aroma at inclusion levels above 10% GR, suggesting that volatile compounds from guava may be transferred to the meat. This highlights the need for further research on the sensory implications of guava residue inclusion beyond color attributes.

The lower cooking weight loss (CWL) observed only for the GR3% diet, compared to GR6% and WB3%, is intriguing. While antioxidants in guava could theoretically help preserve cellular integrity, the effect was not dose-dependent. This specific result may be due to a complex interaction between fiber type, level, and their impact on muscle structure and protein matrix, warranting further investigation. Vieira et al. (2023) found no significant effects of guava residue on cooking loss or water-holding capacity, suggesting that these parameters are more resistant to dietary modulation under isoenergetic and isoprotein conditions. Oliveira et al. (2018) also reported no significant differences in pH or colorimetry, reinforcing that the physical properties of meat are less responsive to dietary fiber than visual attributes.

Regarding the intestinal microbiota, the numerical trend toward lower thermotolerant coliform counts observed in NDF-supplemented groups, particularly in WB6% and GR3% at 28 days and in all supplemented groups at 70 days, aligns with the hypothesis that dietary fiber may modulate the cecal bacterial population. Although inferential analysis was not possible due to sample pooling, these findings are consistent with the mechanisms described by Jha et al. (2019) and Singh and Kim (2021), who reported that fermentable fibers promote the production of short-chain fatty acids, reduce intestinal pH, and create an environment unfavorable for pathogenic bacteria such as Escherichia coli. Oliveira et al. (2018) also observed improvements in ileal villus: crypt ratio with guava by-product inclusion, suggesting enhanced intestinal health, which may indirectly contribute to a more balanced microbiota. Further studies with individual sampling are warranted to confirm these observations.

In conclusion, while NDF supplementation at the tested levels did not enhance the growth performance of slow-growing broilers under the good hygienic conditions of this trial, it yielded specific benefits. The use of guava residue at 6% of NDF improved meat color and sensory preference, and both fiber sources showed a numerical trend toward reducing cecal thermotolerant coliforms. These findings support the potential of guava residue as a functional ingredient for meat quality enhancement in poultry diets.

## Conclusion

Based on the results of this study, the inclusion of 3% NDF from guava residue (GR3%) is recommended for slow-growing broilers in the initial phase (1–28 days), as it maintained performance comparable to the control diet while other NDF-supplemented diets impaired it.

In the growth/finishing phase (29–70 days), NDF supplementation did not affect productive performance. However, the inclusion of 6% NDF from guava residue (GR6%) significantly improved the visual appeal (appearance and color) of breast meat, which translated into the highest consumer preference.

These findings demonstrate that guava residue, used at specific levels for each growth phase, serves as a functional fiber source. It can maintain early growth while enhancing a key sensory attribute linked to consumer acceptance in the final product, adding value to poultry production.

## Data Availability

No applicable.

## Code availability

No applicable.
